# Molecular Engineering of Multi‐Site Small‐Molecule Chelation for Highly Efficient and Stable n‐i‐p Perovskite Solar Cells

**DOI:** 10.1002/advs.78721

**Published:** 2026-11-09

**Authors:** Xinbao Lin, Xiangyang Zhang, Guanghao Meng, Xiaozheng Zhao, Yanan Liu, Tianci An, Min Li, Wenya Zhao, Miaoran Zhang, Linfeng Cui, Taoyu Weng, Yantao Shi, Bo Liu

**Affiliations:** ^1^ Hebei Technology Innovation Center For Energy Conversion Materials and Devices Hebei Engineering Research Center of Thin Film Solar Cell Materials and Devices National Demonstration Center For Experimental Chemistry Education College of Chemistry and Material Science Hebei Normal University Shijiazhuang China; ^2^ State Key Laboratory of Fine Chemicals School of Chemistry Frontier Science Center For Smart Materials Dalian University of Technology Dalian China; ^3^ Yunnan Key Laboratory of Modern Separation Analysis and Substance Transformation College of Chemistry and Chemical Engineering Key Laboratory of Education Ministry For Advance Technique and Preparation of Renewable Energy Materials Yunnan Normal University Kunming China

**Keywords:** hole transport layer, ions migration, p‐doping, perovskite solar cells, Spiro‐OMeTAD

## Abstract

Li‐TFSI‐doped Spiro‐OMeTAD is the benchmark hole‐transporting material for high‐performance n‐i‐p perovskite solar cells (PSCs). Yet its intrinsic instability, arising from Li^+^ migration, has long been regarded as an inevitable trade‐off for achieving high efficiency. Despite extensive efforts, no alternative dopant system has yet fully matched the Li‐TFSI/*t*‐BP system in simultaneously achieving high doping efficiency and well‐established processability. Instead, these alternatives often sacrifice efficiency for improved stability, or they are hampered by synthetic complexity that precludes large‐scale application. Here we design a multifunctional small‐molecule chelator, TPABT3, with multiple coordination sites to immobilize Li^+^ ions and stabilize doped Spiro‐OMeTAD. This molecule concurrently suppresses ion migration, induces ordered molecular packing, and enhances p‐type doping efficiency, while optimizing energy‐level alignment at the perovskite/HTL interface. The resulting two‐step‐fabricated FAPbI_3_ devices deliver an outstanding power conversion efficiency of 26.03% with negligible hysteresis. Moreover, the PSCs retain over 90% of their initial efficiency after 1000 h under ISOS‐L‐1 aging conditions. This work establishes multi‐site molecular chelation as an effective, generalizable strategy for achieving highly efficient and stable n‐i‐p perovskite photovoltaics.

## Graphical abstract

Li^+^ ion migration in Li‐TFSI‐doped Spiro‐OMeTAD impairs efficiency and device stability. Rather than replacing Li^+^, a multi‐site chelator is designed and introduced into Spiro‐OMeTAD to immobilize Li^+^ ions with high affinity. The synergistic effects of suppressing ion migration, inducing ordered molecular packing, boosting p‐doping efficiency, and optimizing interface energetics enable an outstanding PCE of 26.03% with long‐term stability.

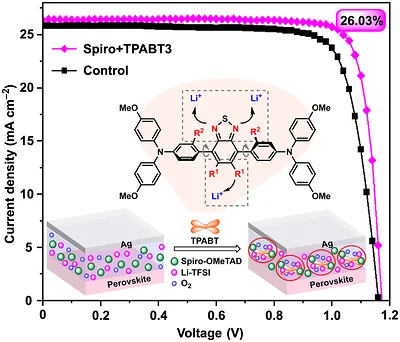

## 1 Introduction

Over the past decade, perovskite solar cells (PSCs) have emerged as leading next‐generation photovoltaic technology, driven by their exceptional optoelectronic properties, solution processability, and an unprecedented pace of efficiency improvements [1, 2, 3, 4, 5]. Among various fabrication routes, we adopt a two‑step sequential deposition method for FAPbI_3_ that decouples nucleation from growth, enabling kinetic control, avoiding the use of antisolvents, and improving reproducibility [6, 7]. In the conventional n‐i‐p device architecture, the hole transport layer (HTL) interfaces with the perovskite, and the back electrode plays a decisive role in both charge extraction and device longevity [8, 9]. To date, 2,2',7,7'‐tetrakis (N,N‐di‐p‐methoxyaniline)‐9,9'‐spirobifluorene (Spiro‐OMeTAD) remains the dominant HTL material for record‐efficiency n‐i‐p devices. However, the performance relies heavily on p‐type doping with lithium bis(trifluoromethanesulfonyl)imide (Li‐TFSI) to achieve adequate conductivity and hole mobility [10, 11]. Simultaneously introducing a processing aid, *tert*‐butylpyridine (*t*‐BP), solely to improve the solubility and uniformity of Li‐TFSI. Yet this conventional doping approach carries a fundamental and unresolved liability. Under operating conditions (illumination, thermal stress, or electrical bias), small Li^+^ ions tend to diffuse across the device stack, triggering interfacial degradation and perovskite lattice distortion, thereby undermining long‐term stability [12, 13, 14]. Furthermore, the slow ambient‐sensitive oxidation of Spiro‐OMeTAD leads to poor device reproducibility [15, 16, 17, 18], while thermal crystallization at elevated temperatures triggers pinhole formation and eventual morphological degradation of the HTL [19, 20].

Extensive efforts have been devoted to circumventing these limitations, including the search for alternative dopants [18, 21, 22, 23] and the development of dopant‐free hole‐transporting materials [24, 25, 26, 27, 28] as replacements for Spiro‐OMeTAD. However, these strategies often fall short in universality or fail to match the peak performance of the classical Li‐TFSI‐doped Spiro‐OMeTAD system. Consequently, a more pragmatic and potentially transformative direction is not to replace Li^+^, but to immobilize it: to preserve the doping capability of Li‐TFSI while neutralizing its detrimental side effects. Recent work has shown that molecular complexation can partially immobilize Li^+^ through coordination chemistry driven by heteroatoms such as oxygen (O) and nitrogen (N). For instance, J. Chen et al. introduced a universal molecular complexation paradigm by embedding bis(2,4,6‐trichlorophenyl)oxalate (TCPO) into the Spiro‐OMeTAD HTL. This additive effectively mitigates Li^+^ ion migration. The Lewis basic C═O units of TCPO establish strong host–guest coordination with Li^+^. Consequently, TCPO‐doped devices achieve a certified power conversion efficiency (PCE) of 25.59% [29]. Similarly, D. S. Kim et al. promoted the formation of uniform HTL films by doping with ethylene carbonate (EC). Owing to the electronegative carbonyl group in EC, a thermodynamically stable EC: Li^+^ coordination complex forms, thereby suppressing ion migration and delivering a PCE of 25.56% [30]. Y. Chen et al. developed a deep eutectic solvent (DES)‐mediated doping strategy employing a binary eutectic mixture of N‐(cyanomethyl)acetamide (NCMA) and Li‐TFSI. Specifically, Li‐O/Li‐N coordination effectively immobilized Li^+^ ions within the Spiro‐OMeTAD matrix. Devices based on this DES strategy achieved a high PCE of 25.02% [31]. While these additives in PSCs mainly rely on carbonyl or amide groups, fundamental coordination chemistry studies on molecular crystals have long established that methoxy groups (‐OMe) on aromatic rings can serve as effective bidentate O‐donor ligands to coordinate Li^+^ and construct ordered ion‐conduction paths [32, 33]. On the other hand, incorporating N‐heterocycles into HTL additives has also proven effective. For instance, Yang et al. designed a composition‐conditioning agent (Spiro‐BD‐2OEG) bearing pyridine rings, where the pyridine N coordinates with Li^+^ while the oligo(ethylene glycol) chains induce ordered packing of Spiro‐OMeTAD [34]. Xiao et al. further demonstrated that an imidazole‐based chelator (HD) with strong dipole moments can coordinate Li^+^ through imidazole N atoms [35]. Although these methods demonstrate promise for improving stability, a fundamental limitation remains: molecular designs are often constrained to a single category or an insufficient number of coordination sites, potentially hindering complete suppression of ion migration or optimal tuning of HTL properties.

Here we report a fundamentally different molecular chelation strategy based on multi‐site coordination. We designed and synthesized a series of small multifunctional molecules (TPABT) that incorporate both nitrogen (N) and methoxy oxygen (O) donors to enable a synergistic multi‐dentate coordination with Li^+^. This multi‐site molecular structure delivers multifaceted improvements: it effectively immobilizes Li^+^ under thermal and light stress, eliminating ion migration and aggregation, thereby stabilizing the perovskite/HTL/electrode interfaces while guiding Spiro‐OMeTAD into a thermodynamically favorable packing characterized by enhanced *π–π* stacking and improved crystallinity; it accelerates the oxidation of Spiro‐OMeTAD, thereby boosting p‐doping efficiency, hole mobility, and conductivity, while simultaneously optimizing interfacial energy level alignment for more efficient hole extraction. These synergistic effects enable Spiro+TPABT3‐based n‑i‑p FAPbI_3_ PSCs to achieve an exceptionally high champion PCE of 26.03%. Furthermore, the TPABT3‐doped unencapsulated device maintains over 90% of its initial PCE after 1000 h of aging under maximum power point tracking (ISOS‐L‐1). Our work highlights the critical strategy of precise molecular design incorporating multi‐site molecular chelation as a generalizable and powerful strategy to unlock the full potential of Li‐doped Spiro‐OMeTAD for both high efficiency and long‐term operational stability in perovskite photovoltaics.

## 2 Results and Discussion

The conventional doping mechanism of Spiro‐OMeTAD is generally considered to proceed via two sequential steps. First, under illumination and in the presence of oxygen, photoinduced electron transfer occurs from Spiro‐OMeTAD to O_2_, generating a transient charge‐transfer complex Spiro‐OMeTAD^•+^O_2_^•−^. Subsequently, Li‐TFSI dissociates to release TFSI^−^, which then displaces O_2_^•−^, yielding a TFSI^−^‐stabilized Spiro‐OMeTAD^•+^TFSI^−^ radical species. This process is concomitantly accompanied by the formation of lithium oxides (Li_x_O_y_) byproducts (Figure 1a). Despite the widespread use of Li‐TFSI as a dopant for Spiro‐OMeTAD in state‐of‐the‐art perovskite solar cells, this system faces ubiquitous challenges, including uncontrolled Li^+^ migration, aggregation‐induced morphological degradation, and inefficient oxidation of Spiro‐OMeTAD, all of which ultimately compromise device stability and performance. To address these issues, we designed and synthesized a series of D‐A‐D molecules TPABT1, TPABT2, and TPABT3 with the same skeleton via Suzuki–Miyaura coupling (Figure 1b and Figure S1), which feature rationally engineered spatial architectures with multiple sites to bind Li^+^ ions, therefore reducing the generation of Li_x_O_y_. The detailed experimental process, nuclear magnetic resonance (NMR), and mass spectrum (MS) data are provided in Figures S33–S43. Among them, the N atom and methoxy (–OMe) side chain on benzothiadiazole can effectively bind with metal ions. The electrostatic potential (ESP) distribution maps (Figure S2) provided initial insights into the potential function of the TPABT molecules. The electron density was predominantly localized on the N atoms of the benzothiadiazole unit and the O atoms of the ─OMe groups, while TPABT3 exhibited the highest electron density due to the presence of more ─OMe groups. This distribution strongly suggests that these atoms can act as Lewis bases, coordinating with Li^+^ ions from Li‐TFSI.

**FIGURE 1:**
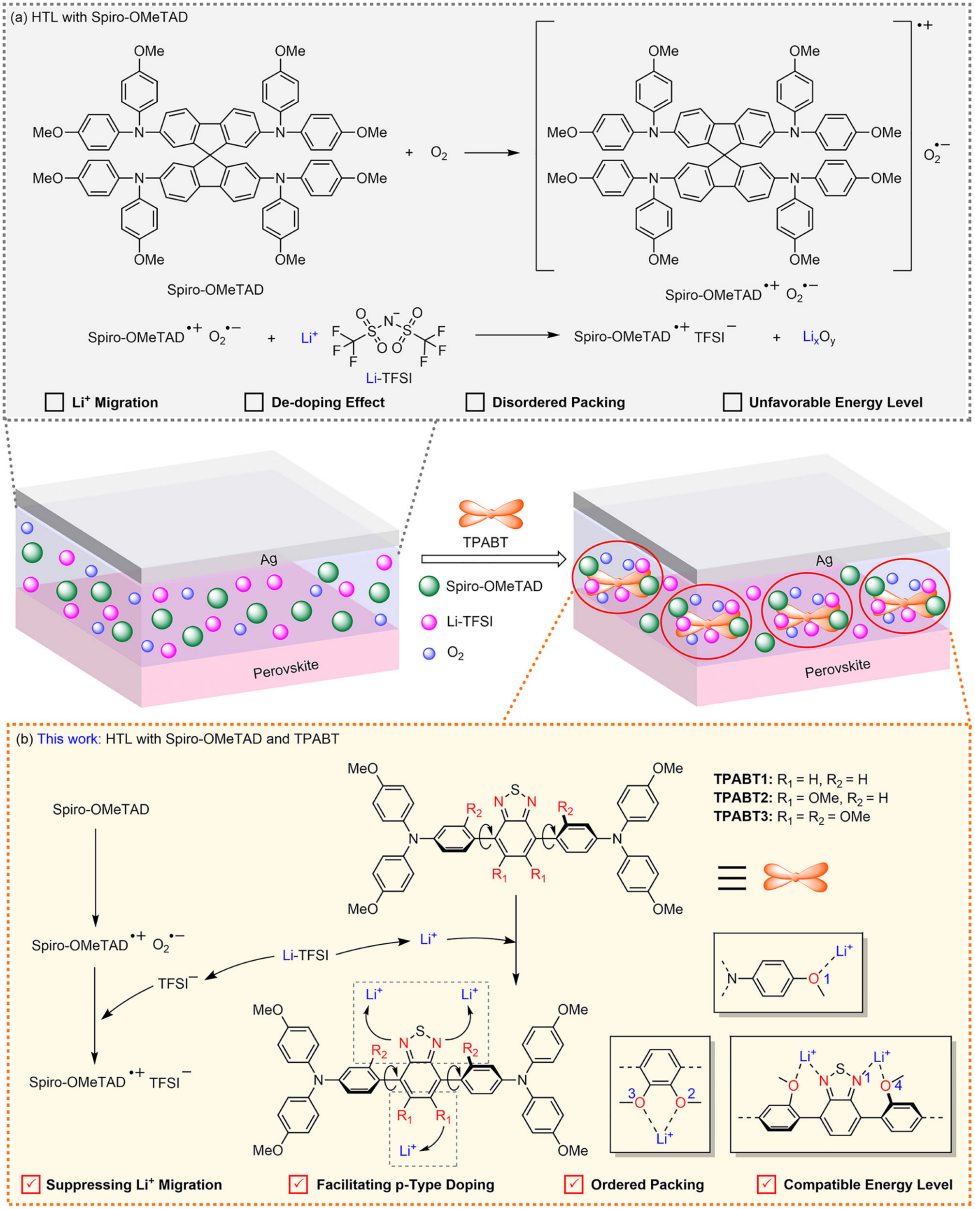
Doping mechanism of control and TPABT‐doped Spiro‐OMeTAD HTLs. (a) Schematic illustration of the mechanism in the conventional doping process (Spiro‐OMeTAD HTLs doped with Li‐TFSI). (b) Schematic illustration of the mechanisms of Li^+^ migration suppression and p‐doping promotion via TPABT‐doped Spiro‐OMeTAD HTLs.

NMR spectroscopy (Figures S35, S38 and S41) was employed to probe the interaction between TPABT and Li^+^ ions; thus, ^7^Li and ^1^H NMR spectra were performed on the Li‐TFSI/TPABT mixtures. As shown in Figure 2a, the Li^+^ resonance shifts upfield from 4.532 to 4.437 ppm, which can be attributed to electron‐density donation from N and O atoms of TPABT3 to the Li^+^ ions, thereby increasing the electron density around Li^+^ and enhancing the shielding effect. Simultaneously, the proton signals of TPABT3 also exhibit an upfield shift (Figure 2b), with the most pronounced change observed for H^1,^ which was ortho to the ─OMe group, shifting upfield by 0.04 ppm. More importantly, solid‐state ^7^Li and ^1^H NMR measurements (Figure S3) exhibit identical variation trends to those observed in the liquid state, confirming that this multi‐site coordination configuration is robustly preserved in the solid‐state HTL matrix after film formation. The Mulliken charge distribution of the atom where C^1^ is in TPABT3 increased from −0.244 to −0.270 upon complexation with Li^+^, indicating greater electron density at this position (Figure 2c). This stems from Li^+^‐driven polarization of the conjugated skeleton, which draws compensatory electron donation from the triarylamine moiety and increases electron density at C^1^. The triarylamine N charge remains nearly unchanged via strong p‐π charge buffering, yielding a distinct phenyl ring charge gradient: proximal rings are more negative (Figure S4). The elevated electron density around the C^1^‐H bond reinforces diamagnetic shielding, accounting for the observed upfield ^1^H NMR shift. Similarly, when TPABT1 and TPABT2 interact with Li‐TFSI, chemical shifts are also observed in both ^7^Li and ^1^H NMR (Figure S5), although the changes are less pronounced than those for TPABT3. These findings indicate that increasing the number of ─OMe groups in the TPABT skeleton further enhances the sites available to interact with Li^+^ ions.

**FIGURE 2:**
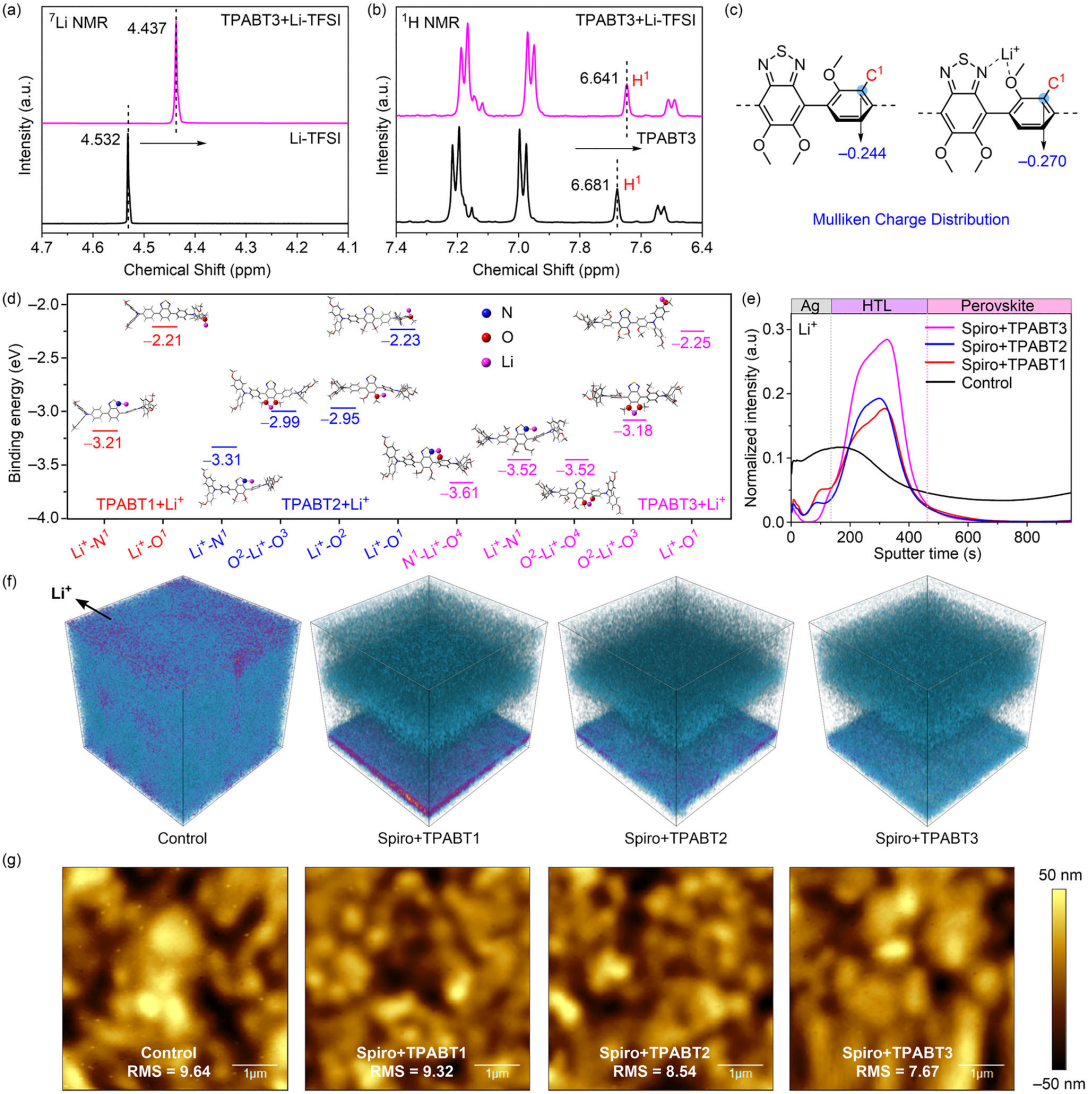
Suppression of Li^+^ migration and aggregation. (a) ^7^Li NMR of the Li‐TFSI solutions without and with TPABT3. (b) ^1^H NMR of the TPABT3 solutions without and with Li‐TFSI. (c) The Mulliken charge distribution of atoms in TPABT3 without and with bound Li^+^ ions. (d) *E*_b_ value of Li^+^ ions with TPABT calculated using density functional theory. (e) TOF‐SIMS depth profile analysis showcasing the distribution of Li^+^ ions based on the control and doped Spiro‐OMeTAD devices. (f) The corresponding 3D rendered overlay images of Li^+^ ions in the control and TPABT‐doped devices. (g) AFM images of control and TPABT‐doped thin films.

Density functional theory (DFT) calculations are used to gain an in‐depth understanding of atomic‐level binding characteristics between Li^+^ ions and TPABT molecules (Figure 2d). According to the ESP analysis, the main binding sites of Li ions are O^1^, O^2^, O^3^, O^4^, and N^1^ (Figure 1b). For TPABT1, there are only two possible binding modes with Li^+^, and the binding energy (*E*_b_) values for Li^+^–O^1^ and Li^+^–N^1^ are −2.21 and −3.21 eV, respectively. Upon introducing ─OMe groups at the corresponding positions, TPABT2 and TPABT3 not only offer additional Li^+^ coordination sites but also exhibit stronger and more diverse binding configurations. In TPABT2, the *E*_b_ values of Li^+^–O^1^ and Li^+^–N^1^ increased, and two new binding modes emerged: Li^+^–O^2^ (*E*_b_ = −2.95 eV) and the bidentate coordination O^2^–Li^+^–O^3^ (*E*_b_ = −2.99 eV), which collectively strengthened the immobilization of Li^+^. For TPABT3, the *E*_b_ values of Li^+^–N^1^ and O^2^–Li^+^–O^3^ were further enhanced to −3.18 and −3.52 eV, respectively. Moreover, two additional bidentate modes, O^2^–Li^+^–O^4^ (*E*_b_ = −3.52 eV) and N^1^–Li^+^–O^4^ (*E*_b_ = −3.61 eV), were formed. The most notable is the N^1^–Li^+^–O^4^ coordination, which exhibited the highest binding energy among all configurations, thermodynamically favorable interactions, and corresponds to the obvious shift of the H^1^ peak in NMR. Among the three molecules, TPABT3 possesses the highest Li^+^ binding affinity, making it the most effective candidate for mitigating Li^+^ migration within the HTLs.

To visually confirm this inhibition of Li^+^ diffusion and the coordination effect of TPABT3, time‐of‐flight secondary ion mass spectrometry (TOF‐SIMS) was performed to characterize the distribution of Li^+^ ions in aged devices. For ease of description, regular doped Spiro‐OMeTAD was referred to as control. The TPABT‐doped solutions and films were referred to as Spiro+TPABT1, Spiro+TPABT2, and Spiro+TPABT3. As shown in Figure 2e,f, a strong Li^+^ signal is observed at both the silver electrode and the perovskite layer in the control device aged at 85°C for 5 days, indicating severe perovskite degradation and significant Li^+^ migration. In sharp contrast, Li^+^ remains largely confined to the TPABT‐doped HTL, whereas only a negligible fraction is detected in other layers. The Li^+^ intensity in the TPABT3‐doped HTL is approximately two orders of magnitude higher than the control sample, which visually demonstrates the significant inhibition of Li^+^ ion diffusion by the TPABT3 molecule, due to its multiple ─OMe groups and strong binding modes.

To correlate the film morphology with Li‐TFSI aggregation, atomic force microscopy (AFM) was performed on the control and doped HTL after aging at 85°C for 5 days (Figure 2g). The root mean square (RMS) roughness values were significantly decreased from 9.64 nm for control samples to 9.32, 8.54, and 7.67 nm for TPABT‐doped samples, respectively. This progressive smoothing indicates more intimate contact between HTL and perovskite, which is beneficial for interfacial carrier transport. Notably, the control Spiro‐OMeTAD film exhibits a pitted surface with shallow defects, attributed to the migration and aggregation of Li‐TFSI within the HTL. These pits nearly disappear upon incorporation of TPABT molecules, indicating suppressed additive aggregation. To gain insight into the chemical origin of this morphological change, X‐ray photoelectron spectroscopy (XPS) measurements were conducted (Figure S6). A strong Li 1s signal at 56.1 eV is evident in the control Spiro‐OMeTAD film. In contrast, the virtual absence of this peak in the TPABT‐doped films demonstrates that TPABT molecules, especially TPABT3 with optimized multi‐binding architecture, can effectively suppress Li^+^ diffusion and aggregation in the HTL.

The incorporation of TPABT molecules effectively passivates the hygroscopic [36, 37, 38] aggregation of Li‐TFSI via robust Li^+^ coordination. Thereby providing an unperturbed environment for the Spiro‐OMeTAD molecules to adopt a more thermodynamically favorable and ordered packing motif during film solidification. This is unequivocally evidenced by the sharper diffraction rings and enhanced crystallinity of doped HTLs observed in Grazing‐incidence wide‐angle X‐ray scattering (GIWAXS) patterns (Figure 3a,b), indicating enhanced intermolecular interaction. Pristine Spiro‐OMeTAD has a disordered structure. After TPABT molecules are incorporated, the diffraction peak at 1.29 Å^−1^ associated with *π–π* stacking becomes more intense, indicating a more ordered arrangement in the doped HTLs. According to the Scherrer equation [39], the calculated crystalline coherence lengths (CCLs) increased from 8.28 Å (control) to 10.17 Å (Spiro+TPABT3), indicating the Spiro+TPABT3 film exhibited long‐range molecular ordering and higher crystallinity. We then investigated the mechanism of TPABT3‐induced Spiro‐OMeTAD organization. In situ absorption spectroscopy was employed to monitor the film crystallization kinetics by tracking the UV–vis absorption edge and intensity. The film crystallization process can be divided into three stages: (I) solvent evaporation, (II) crystallite growth, and (III) film formation. During stage I, the absorption spectra of the two samples (Spiro‐OMeTAD and Spiro+TPABT3) remained almost unchanged, indicating that no organization occurred during this stage because the solvent evaporated at a high rate and could only drive the Spiro‐OMeTAD molecules closer to each other. During stage II, the Spiro+TPABT3 sample showed a relatively longer time (1.165 s) than the control (0.804 s), which was used to monitor the film crystallization process; this also suggested an extended crystallite growth time of the Spiro+TPABT3 film compared to that of the control film (Figure S7). These results suggest that the addition of TPABT3 could effectively prolong the Spiro‐OMeTAD crystallization process, which is beneficial for providing sufficient time for molecular organization. Furthermore, the binding energy between TPABT3 and Spiro‐OMeTAD (*E*_b_ = −0.26 eV) is larger than the self‐binding energy between two Spiro‐OMeTAD molecules (*E*_b_ = −0.23 eV). This strong intermolecular affinity provides a robust thermodynamic driving force for TPABT3 to actively coordinate with Spiro‐OMeTAD molecules(Figure S8). This enhanced molecular ordering is expected to facilitate charge carrier transport. Energy‐dispersive spectroscopy (EDS) of doped HTL further indicates uniform elemental distribution of TPABT within the Spiro‐OMeTAD matrix, ruling out localized aggregation that could compromise film integrity (Figure S9). Consistently, the reduced Urbach energy (*E*_u_) of the doped HTL indicates suppressed energetic disorder within the sub‐bandgap states [40] (Figure S10).

**FIGURE 3:**
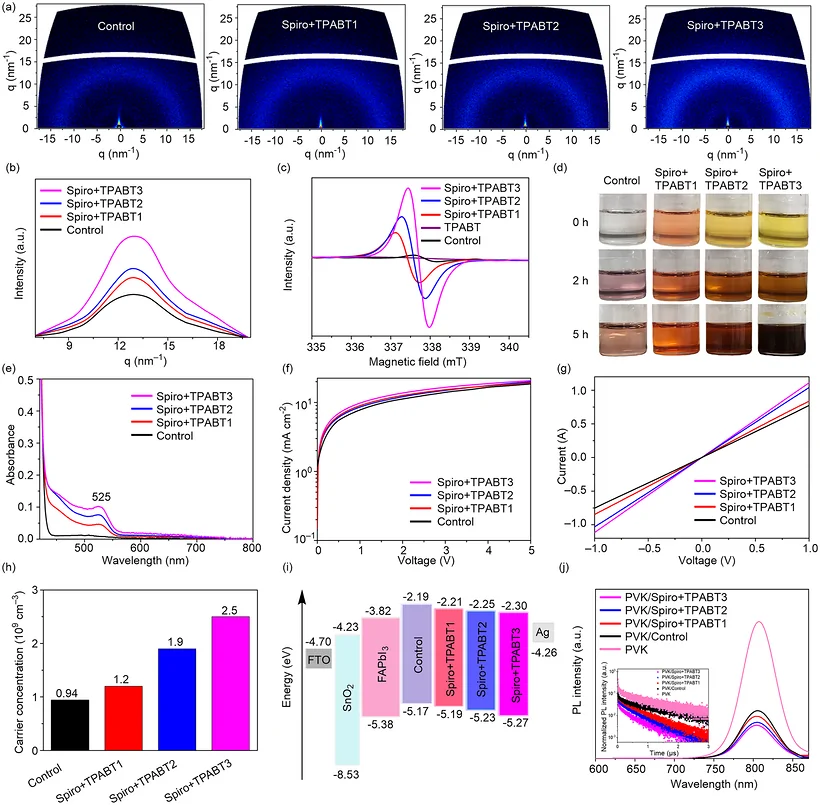
Stacking order regulation of Spiro‐OMeTAD and facilitated p‐type doping by TPABT molecules. (a) 2D GIWAXS pattern of control and doped Spiro‐OMeTAD thin films. (b) Comparison of 1D GIWAXS profiles of control and doped Spiro‐OMeTAD thin films. (c) EPR spectra of control and doped Spiro‐OMeTAD solutions. (d) Comparison of the color changes of control and doped Spiro‐OMeTAD solutions before and after 2 and 5 h of oxidation in ambient air. (e) UV–vis absorption spectra in chlorobenzene solutions of control and doped Spiro‐OMeTAD. (f) *I‐V* curves of ITO/control and doped Spiro‐OMeTAD/Au devices. (g) *I‐V* curves of ITO/PEDOT: PSS/control and doped Spiro‐OMeTAD/Au devices. (h) The carrier concentration of control and doped Spiro‐OMeTAD films. (i) Energy level diagrams of PSCs. (j) Steady‐state PL emission spectra and TRPL spectra of the PVK with control and doped Spiro‐OMeTAD thin films.

While the enhanced molecular ordering induced by Li^+^ coordination facilitates bulk hole transport, the presence of TPABT may also promote the formation of stable Spiro‐OMeTAD radical cations. To explore this possibility, we employed electron paramagnetic resonance (EPR) spectroscopy to assess the p‐doping efficiency. (Figure 3c). The Spiro‐OMeTAD doped with TPABT showed notable paramagnetic peaks at 336–339 mT compared with the control sample. The crystal data for TPABT3 exclude an open‐shell configuration of the molecule itself (Figure S11). Correspondingly, this agrees with the EPR results, as neither the Spiro‐OMeTAD + TPABT mixture nor the TPABT + Li‐TFSI mixture gives any EPR signal. (Figure S12). This confirms that the paramagnetic signal obtained above indeed originates from Spiro‐OMeTAD^•+^. The above results confirm that TPABT molecules did not directly participate in the p‐type doping process, but indirectly promoted the oxidation of Spiro‐OMeTAD. Meanwhile, we found that Spiro‐OMeTAD solution doped with TPABT turned dark in a shorter period (Figure 3d). This process was visually confirmed by the UV–vis absorption spectrum (Figure 3e). After being placed in ambient air for 2 h, the control sample spectrum showed no obvious changes, while Spiro‐OMeTAD doped with TPABT exhibited a characteristic absorption band at ∼525 nm, attributed to the production of Spiro‐OMeTAD^•+^TFSI^−^, indicating accelerated oxidation of Spiro‐OMeTAD. The UV–vis absorption spectra (Figures S13 and S14) of the individual TPABT1‐3 solutions and their respective mixtures with Li‐TFSI reveal that the characteristic absorption peaks of these control solutions are located entirely in the shorter wavelength region, with no absorption observed at 525 nm. Doping with TPABT1, TPABT2, and TPABT3 successively increases the EPR intensity and absorbance at 525 nm, indicating that the introduction of ─OMe units promotes the formation of Spiro‐OMeTAD^•+^TFSI^−^. Furthermore, under oxygen‐free conditions, no radical signals were detected via EPR spectroscopy, UV–vis absorption measurements, or solution color change observations, indicating that TPABT3 cannot facilitate the oxidation of Spiro‐OMeTAD in the absence of oxygen (Figures S15–S17). Based on the experimental results, we propose a chemical mechanism in which TPABT acts as a Li^+^‐chelating promoter. (Figure 1b). TPABT coordinates with Li^+^ to facilitate the dissociation of TFSI^−^ anions from Li‐TFSI. The increased TFSI^−^ anion concentration drives the oxidation equilibrium of Spiro‐OMeTAD forward, favoring the conversion of unstable Spiro‐OMeTAD^•+^O_2_^•−^ into detectable Spiro‐OMeTAD^•+^TFSI^−^. Thus, TPABT indirectly participates in the p‐type doping process and accelerates the oxidation of Spiro‐OMeTAD.

To directly evaluate the actual p‐type doping effect, the hole mobility and conductivity were measured. Hole mobility (*µ*_h_) determined using the space‐charge‐limited‐current (SCLC) method, increased from 1.73 × 10^−3^ cm^2^ V^−1^ s^−1^ for the control film to 1.80 × 10^−3^, 1.88 × 10^−3^ and 1.97 × 10^−3^ cm^2^ V^−1^ s^−1^ for the Spiro+TPABT1, Spiro+TPABT2, and Spiro+TPABT3 films, respectively (Figure 3f). Correspondingly, electrical conductivity was enhanced (Figure 3g), with values of 1.88 × 10^−4^, 2.34 × 10^−4,^ and 2.50 × 10^−4^ S cm^−1^ for the TPABT1, TPABT2, and TPABT3‐doped films, compared to the control film (1.74 × 10^−4^ S cm^−1^). This improvement is attributed to the elevated concentration of radicals generated by Spiro‐OMeTAD, which not only injects positive charge carriers but also fills deep trap states, thereby enhancing carrier mobility. Hall effect measurements were further conducted to analyze the carrier concentration in control and doped Spiro‐OMeTAD. It is found that the average carrier concentrations were 1.2 × 10^9^, 1.9 × 10^9^, and 2.5 × 10^9^ cm^−3^ for TPABT1, TPABT2, and TPABT3‐doped HTL (Figure 3h and Figure S18), which are higher than the control HTL (0.94 × 10^9^ cm^−3^). This systematic enhancement demonstrates that incorporating TPABT‐based dopants, particularly TPABT3, significantly improves the photogenerated hole transport properties of Spiro‐OMeTAD.

Given that p‐doping shifts the Fermi level toward the HOMO edge in organic semiconductors, the pronounced enhancement in hole mobility implies a corresponding deepening of the effective work function. We therefore performed ultraviolet photoelectron spectroscopy (UPS) and UV–vis spectroscopy to directly probe the energy‐level alignment at the perovskite/HTL interface (Figures S19 and S20). The detailed energy level parameters are summarized in Table S2. The Fermi levels of the control, TPABT1, TPABT2, and TPABT3‐doped HTLs were determined to be −4.15, −4.30, −4.33, and −4.36 eV, respectively. The incorporation of TPABT shifts the Fermi level downward in the Spiro‐OMeTAD‐based HTL, indicating enhanced hole transport efficiency and p‐type doping in HTLs. As shown in Figure 3i, compared to undoped Spiro‐OMeTAD, the valence band maximum (VBM) values for TPABT1‐, TPABT2‐, and TPABT3‐doped HTLs increased to −5.19, −5.23, and −5.27 eV, respectively, from a baseline of −5.17 eV. This shift demonstrates improved alignment with the perovskite VBM (−5.38 eV), thereby boosting open‐circuit voltage (*V*_OC_). To further probe the effect on hole extraction dynamics, steady‐state photoluminescence (PL) and time‐resolved photoluminescence (TRPL) spectra were obtained. As shown in Figure 3j and Table S3, the doped Spiro‐OMeTAD exhibits stronger PL intensity quenching compared to the control Spiro‐OMeTAD. The TRPL decay curves show that the perovskite film without HTL exhibits an average carrier lifetime of 1.27 µs. This lifetime decreases significantly to 947.61, 730.44, 433.96, and 332.49 ns for perovskite/control, perovskite/Spiro+TPABT1, perovskite/Spiro+TPABT2, and perovskite/Spiro+TPABT3 samples, respectively. These results, together with the improved energy‐level alignment and enhanced electrical properties of the HTL, indicate that the enhanced PL quenching is dominated by accelerated hole extraction rather than by defect‐mediated non‐radiative recombination.

As discussed above, the TPABT3‐doped Spiro‐OMeTAD combines superior film morphology, enhanced hole mobility, and excellent charge extraction capability, rendering it a highly promising HTL. To evaluate the actual photovoltaic performance, n‐i‐p structured PSCs were fabricated with the architecture FTO/SnO_2_/FAPbI_3_/HTL/Ag (Figure 4a). The optimal doping concentration for TPABT dopants was determined to be 5 mol% (Figure S21 and Table S4). As shown in Figure 4b, the control device exhibited a short‐circuit current density (*J*_SC_) of 25.85 mA cm^−2^, *V*_OC_ of 1.16 V, fill factor (*FF*) of 79.45%, and a PCE of 23.85%. PCEs for Spiro+TPABT1 and Spiro+TPABT2 based devices improved to 24.79% (*J*_SC_ = 26.13 mA cm^−2^, *V*_OC_ = 1.16 V, *FF* = 81.64%) and 25.42% (*J*_SC_ = 26.24 mA cm^−2^, *V*_OC_ = 1.16 V, *FF* = 83.35%), respectively. Comparatively, the Spiro+TPABT3‐based device achieved a champion PCE of 26.03%, with an improved *J*_SC_ of 26.39 mA cm^−2^, excellent *V*_OC_ of 1.17 V, and high *FF* of 84.08%. This performance highlights the beneficial effect of increasing ─OMe substitution, attributable to more efficient charge transport dynamics, as evidenced by improvements in *V*_OC_, *J*_SC_, and *FF* parameters. Furthermore, the hysteresis index decreased to 1.8%, 1.2%, and 0.4% for Spiro+TPABT1‐, Spiro+TPABT2‐, and Spiro+TPABT3‐based devices, respectively, compared to 2.3% for the control device (Figure S22 and Table S5), indicating effective suppression of Li^+^ ion migration and enhanced charge extraction. Integrated *J*_SC_ values derived from the external quantum efficiency (EQE) spectrum of the Spiro+TPABT1 (25.43 mA cm^−2^), Spiro+TPABT2 (25.64 mA cm^−2^), and Spiro+TPABT3 (25.91 mA cm^−2^) devices closely matched the *J‐V* characteristics (Figure 4c). The enhanced EQE in the long‐wavelength range of 650–780 nm is primarily attributed to the optimized energy‐level arrangement at the interface between perovskite and doped HTL [41]. Compared with the photovoltaic parameters in Figure 4d and Table S6, our TPABT3‐doped two‐step regular PSC achieves a PCE of 26.03% with a *J*_SC_ exceeding that of a device exhibiting the highest reported PCE to date (26.16%), suggesting that enhanced p‐doping facilitated by TPABT3 contributes to more efficient hole extraction and collection. Statistical analysis of 20 devices confirmed the superior reproducibility of doped HTLs (Figure S23). Figure S24 shows stable power output (SPO) at the maximum power point of devices for 300 s. Compared with the control device (22.89%), the TPABT1, TPABT2, and TPABT3‐doped PSCs achieved higher SPO of 23.59%, 24.26%, and 25.26%, respectively. These PCE improvements resulted from expedited p‐type doping, increased hole mobility, and improved energy level matching mediated by TPABT molecules.

**FIGURE 4:**
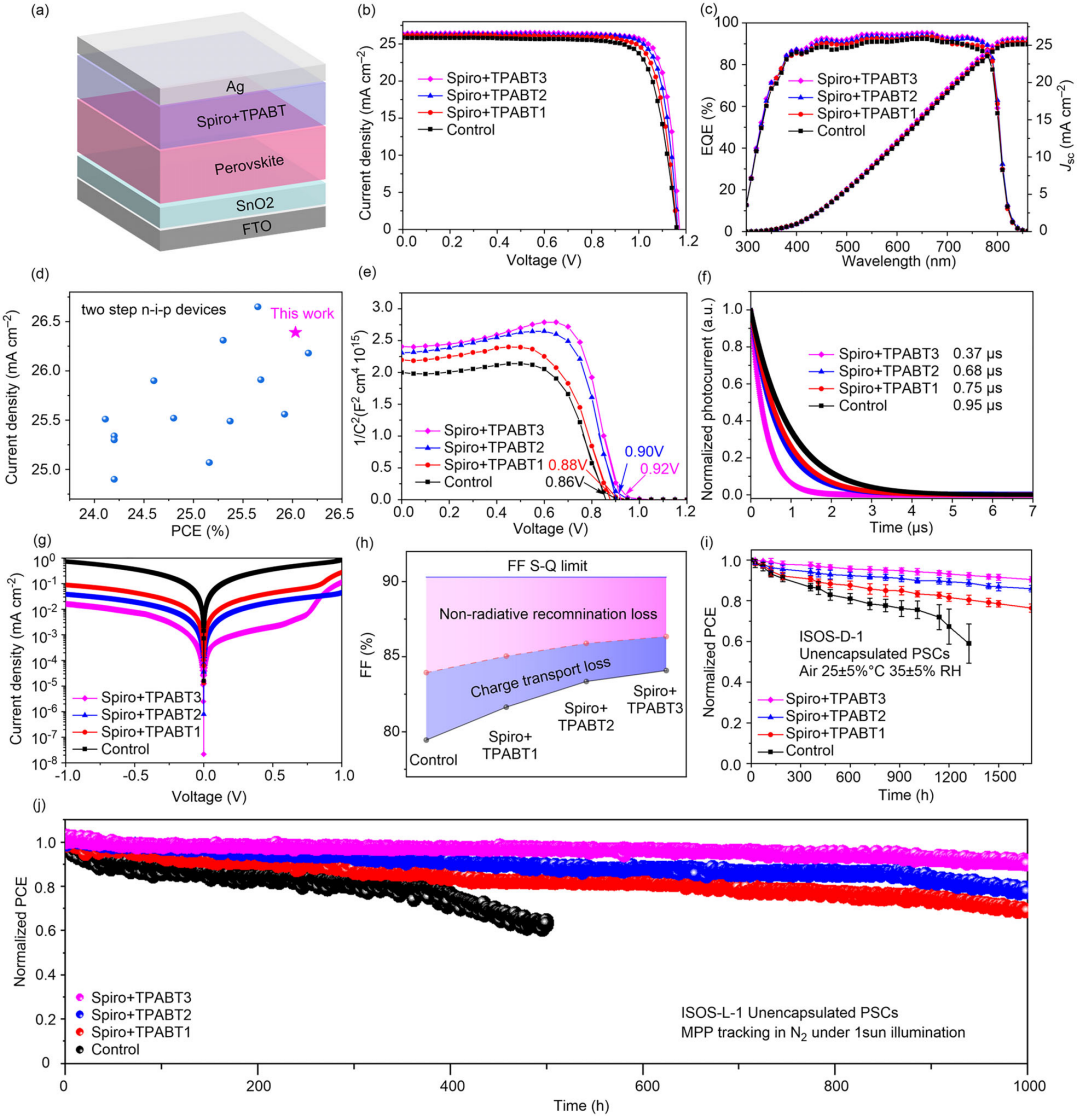
Photovoltaic performance and long‐term stability of devices. (a) The device structure of PSCs. (b) Current density‐voltage characteristics of the control and doped Spiro‐OMeTAD champion devices. (c) EQE spectra and the integrated *J*sc. (d) PCE comparison between this work and reported high‐efficiency two‐step regular FAPbI_3_ PSCs. (e) Mott–Schottky plots of control and doped devices. (f) The TPC decay curves of control and doped devices. (g) Dark *I‐V* curves of control and doped devices. (h) The device *FF* limitation composed of non‐radiative loss and charge transport loss. (i) Storage stability of control and doped devices. (j) Operational stability of the unencapsulated control and doped devices at MPP under continuous 1 sun irradiation.

To elucidate the mechanism of device efficiency improvement, electrochemical and optoelectronic characterization were performed to investigate charge extraction and carrier recombination. The increase in *V*_OC_ is primarily governed by reduced carrier recombination; to probe recombination mechanisms, the relationship between *V*_OC_ and light intensity (*P*_light_) was examined. The ideality factor for the control was 1.64 kT/q, whereas for Spiro+TPABT1‐, Spiro+TPABT2‐, and Spiro+TPABT3‐based devices, it was reduced to 1.59, 1.47, and 1.42 kT/q, respectively, suggesting effective suppression of non‐radiative recombination (Figure S25). Complementing this, the TPABT3‐doped device exhibited a higher built‐in potential (*V*_bi_) of 0.92 V during the Mott–Schottky characteristics (Figure 4e), compared to 0.86, 0.88, and 0.90 V for control, Spiro+TPABT1‐, and Spiro+TPABT2‐based devices, respectively. The enhanced *V*_bi_ provided an enhanced driving force for the separation of photogenerated charge carriers and mitigated non‐radiative recombination, thereby contributing to high *V*_OC_. Transient photovoltage (TPV) decay measurements (Figure S26) corroborate these findings: the TPABT3‐doped device exhibits a longer carrier lifetime relative to the control, confirming that non‐radiative recombination is successfully suppressed. The improvement in *J*_SC_ is attributed to faster charge extraction and more efficient carrier collection. To directly assess charge extraction kinetics, transient photocurrent (TPC) measurements were performed (Figure 4f). The carrier decay lifetime is substantially reduced from 0.95 µs (control) to 0.75, 0.68, and 0.37 µs for the TPABT1, TPABT2, and TPABT3‐doped devices, respectively. This marked acceleration demonstrates that the doped HTLs enable much faster extraction and transport of photogenerated carriers, directly boosting *J*_SC_. The enhanced charge collection efficiency was further corroborated by the power‐law correlation between *J*_SC_ and light intensity (Figure S27). For TPABT1, TPABT2, and TPABT3‐doped devices, the α values were fitted to 0.95, 0.97, and 0.98, respectively, which are closer to 1 than the control device (α = 0.93). These results indicate rapid charge collection and minimize carrier accumulation at the perovskite/doped HTL interface. The reduced trap density also facilitates more efficient charge collection. Space‐charge‐limited current (SCLC) measurements (Figure S28) reveal that all doped devices exhibit lower trap densities, decreasing from 2.47 × 10^15^ cm^−3^ (control) to 1.45 × 10^15^ cm^−3^ (TPABT3), suppressing trap‐assisted recombination during charge transport, thereby contributing to the enhanced *J*_SC_. The enhancement in *FF* is primarily linked to improved charge transport and reduced shunting pathways. Electrochemical impedance spectroscopy (EIS) was employed to analyze carrier transport dynamics (Figure S29). The high‐frequency resistance in the Nyquist plot corresponds to the charge‐transport resistance (*R*_ct_) within the device. Compared to the control device (*R*_ct_ = 7.02 kΩ), the *R*_ct_ values for TPABT1, TPABT2, and TPABT3‐doped devices decreased to 6.12, 5.41, and 4.57 kΩ, respectively, indicating a reduced hole‐transfer barrier at the perovskite/HTL/Ag interface and correlating with *FF* enhancement. In parallel, the dark *I‐V* curves (Figure 4g) demonstrated lower leakage currents for doped devices, with the TPABT3‐doped device exhibiting the lowest, confirming enhanced shunt resistance and suppressed recombination. Finally, the *FF* loss between the Shockley–Queisser limit (*FF*_S‐Q limit_) and measured values (*FF*_real_) is attributed to non‐radiative recombination and charge‐transport losses. In contrast to the control device, both losses decreased markedly, contributing to the enhanced *FF* of doped devices (Figure 4h). In conclusion, TPABT dopants, particularly TPABT3, comprehensively enhance HTL performance by improving conductivity and energy‐level alignment via p‐type doping, optimizing charge transport pathways through promoting molecular ordering, and reducing interfacial defect‐assisted recombination. Taken together, superior PCE, characterized by high *V*_OC_, *J*_SC_, and *FF*, was observed in TPABT3‐doped devices.

The long‐term operational stability of PSCs remains a critical barrier to commercialization. The durability of devices was systematically assessed under various accelerated aging conditions, revealing that TPABT dopants, particularly TPABT3, confer exceptional robustness to the devices. Under ISOS‐D‐1 protocols (25°C, 35% RH, air), unencapsulated TPABT3‐doped devices retained 90.5% of initial PCE after 1700 h, significantly outperforming TPABT1 (85.9%) and TPABT2 (76.4%) doped devices (Figure 4i). This enhancement is attributed to the improved HTL stability, which effectively mitigates moisture infiltration into the perovskite lattice. The moisture stability of these unencapsulated PSCs was further evaluated in ambient air. After aging at 85% RH for 800 h, the TPABT1‐, TPABT2‐, and TPABT3‐doped devices maintained 78.6%, 84.2%, and 91.4% of their initial efficiency, respectively (Figure S30). This enhancement was attributed to the hydrophobic properties of TPABT, as evidenced by increased water contact angle (WCA) for doped HTL films (Figure S31). Notably, the WCA values of the doped HTL films increased from 80° (Spiro+TPABT1) to 87° (Spiro+TPABT3), surpassing that of the pristine Spiro‐OMeTAD (73°). This confirms that the moisture tolerance of Li‐TFSI‐doped Spiro‐OMeTAD film is effectively improved upon the addition of TPABT. Subsequently, the thermal stability of PSCs was evaluated through thermal stress experiments at 85°C under an N_2_ atmosphere with ambient light. The TPABT3‐doped device exhibits a 20.8% efficiency loss after 800 h, whereas the TPABT1‐ and TPABT2‐doped devices degrade to 61.2% and 73.5% of the original PCE, respectively (Figure S32). These results indicate that TPABT dopants significantly enhance the operational stability of devices. The markedly improved stability of the doped devices is attributed to suppressed Li^+^ migration and enhanced crystallization of the HTL. Additionally, maximum power point tracking under continuous one‐sun illumination further confirms the superior operational stability of Spiro+TPABT3‐based PSCs, with 90.1% of its initial PCE retained after 1000 h; the control device maintains only 63.3% of the original efficiency after 500 h (Figure 4j). Overall, the TPABT doping strategy simultaneously enhances photovoltaic performance and long‐term durability by inhibiting Li^+^ migration and facilitating p‐type doping.

## 3 Conclusion

In summary, we developed a series of multifunctional organic small‐molecule dopants (TPABT) featuring a rationally designed spatial arrangement of methoxy groups that establishes an effective multi‐site cooperative chelation strategy, efficiently suppressing Li^+^ ion migration and aggregation. The dopant, particularly TPABT3, markedly promotes ordered packing and accelerates the p‐doping of Spiro‐OMeTAD, thereby enhancing charge‐carrier mobility and optimizing energy‐level alignment at the critical perovskite/HTL interface. Consequently, the TPABT‐doped regular FAPbI_3_ PSCs achieve an outstanding champion PCE of 26.03% with negligible hysteresis. Furthermore, these devices retain 90% of initial PCE after 1000 h under continuous one‐sun illumination. This work successfully demonstrates that engineering multi‐site Li^+^ ion chelators is a powerful and generalizable strategy to concurrently address the challenges of efficiency and stability in Spiro‐OMeTAD‐based perovskite photovoltaics.

## 4 Experimental Section

### 4.1 Materials

Fluorine‐doped tin dioxide (FTO, 7 Ω sq^−1^) glass substrates, methylammonium chloride (MACl, 99.9%), 2‐phenylethylamine hydroiodide (PEAI, 99.9%), N, N‐dimethylformamide (DMF, 99.9%), dimethyl sulfoxide (DMSO, 99.9%), isopropanol (IPA, 99.9%), Chlorobenzene (CB, 99.5%) and acetonitrile (ACN, 99.5%) were purchased from Advanced Electron Technology Co., Ltd. Lead(II) iodide (PbI_2_, 99.9%, trace metal basis) was purchased from Tokyo Chemical Industry (TCI). Formamidinium iodide (FAI, 99.8%) was purchased from Greatcell Solar Materials. 2,2′,7,7′‐tetrakis (N, N‐di‐pmethoxyphenylamine)−9, 9′‐spirobifluorene (Spiro‐OMeTAD, 99.8%) was purchased from Borun New Material Technology Co., Ltd. Bis(trifluoromethane) sulfonimide lithium salt (Li‐TFSI, 99.9%) was purchased from Aladdin. The SnO_2_ colloid precursor (tin (IV) oxide, 12% in H_2_O colloidal dispersion) was purchased from Xi'an Yuri Solar Co., Ltd. Silver electrode material (99.9%) and 4‐tert‐butylpyridine (TBP) were purchased from Yingkou OPV Tech New Energy Co., Ltd. 2‐Bromo‐5‐iodoanisole (98%) and 4,7‐Dibromo‐2,1,3‐benzothiadiazole (98%) were purchased from Energy Chemical. 4,7‐Dibromo‐5,6‐dimethoxybenzo[c] [1, 2, 5] thiadiazole (98%) and 4‐Methoxy‐N‐(4‐methoxyphenyl)‐N‐(4‐(4,4,5,5‐tetramethyl‐1,3,2‐dioxaborolan‐2‐yl) phenyl) aniline (98%) were purchased from SunaTech Inc. All chemicals and solvents were used as received without further purification.

### 4.2 Device Fabrication

The FTO glass substrates (1.9 × 1.9 cm^2^) were cleaned by sonication with detergent, deionized water, isopropanol, and acetone for 20 min each, followed by UV ozone for 15 min. The precursor of the SnO_2_ ETL was prepared by mixing the SnO_2_ colloid precursor with ultrapure water at a volume ratio of 1:4. A total of 90 µL SnO_2_ colloidal solution was spin‐coated onto the cleaned FTO substrates at 3000 rpm for 45 s, followed by drying at 150°C for 30 min in ambient air. The perovskite layer was prepared by the two‐step method. First, 1.5 M PbI_2_ was dissolved in a mixed anhydrous solvent (DMF: DMSO = 9: 1) by stirring at 70°C for 1 h. Second, 90 mg FAI and 12 mg MACl were dissolved in 1 mL IPA solvent by magnetic stirring for 1 h. In an N_2_ glove box, 30 µL PbI_2_ solution was spin‐coated at a rate of 1500 rpm for 30 s, followed by thermal annealing at 70°C for 40 s; then the 110 µL organic salt precursor solution was spin‐coated onto PbI_2_ at 1700 rpm for 25 s and annealed at 90°C for 25 s. Subsequently, the perovskite films were annealed at 150°C for 15 min in the ambient atmosphere (RH ≈ 30%–40%). For PEAI post‐treatment, 100 µL PEAI solution (1.3 mg mL^−1^ in IPA) was spin‐coated onto perovskite films at 5000 rpm for 30 s without additional thermal annealing. The Spiro‐OMeTAD solution was prepared by dissolving 17.5 µL of Li‐TFSI solution (520 mg/mL Li‐TFSI in acetonitrile), 72.3 mg of Spiro‐OMeTAD, and 28.8 µL of *t*‐BP in 1000 µL CB. For the doped Spiro‐OMeTAD solution, different concentrations (2, 5, and 10 mol%) of TPABT1, TPABT2, and TPABT3 were added to the Spiro‐OMeTAD system. Then, 30 µL of HTL solution was deposited on the as‐prepared perovskite substrates at 3000 rpm for 30 s. Finally, a 76 nm Ag electrode was thermally evaporated under high vacuum conditions (< 5 × 10^−4 ^Pa) using a shadow mask.

### 4.3 Characterization

The ^1^H and ^13^C proton nuclear magnetic resonance (NMR) spectra were recorded on a WIPM‐NMR‐400 spectrometer with the chemical shifts referenced to trimethylsilyl. ^7^Li NMR was measured by JNM‐ECZ600R/S1. High‐resolution mass spectra (HRMS) were performed on an Agilent 1290InfinityII‐6545 with methanol as solvent. X‐ray single‐crystal diffraction data were collected with a Bruker D8 Venture and Pilatus detector d with Cu radiation. UV–vis absorption spectra of solutions and films were measured using Agilent Cary60 and Cary5000 spectrophotometers. Electron Paramagnetic Resonance (EPR) spectra were measured using a Freiberg Instruments Germany MS5000 spectrometer. Depth profiling of PSCs was analyzed by time‐of‐flight secondary ion mass spectrometry (TOF‐SIMS) using an IONTOF M6 instrument from IONTOF GmbH, using a 30 keV Bi_3_^+^ primary ion source with an ion current of 0.3 pA and a 1 keV Ar sputter ion gun. Atomic force microscopy (AFM) analysis was performed using MFP‐3D Origin (Oxford Instruments Asylum Research Inc.). X‐ray photoelectron spectra (XPS) characterizations were carried out on a Thermo Fisher ESCALAB QXi system with an Al Kα source, and the core‐level C 1s (C─C bond at 284.8 eV) was used as the reference to calibrate the energy position. UPS measurements were conducted using a Thermo Fisher Scientific ESCALAB XI^+^ with a non‐monochromated He Iα photon source (*hν* = 21.22 eV). A bias of −5 V was applied to the sample during UPS measurements. Grazing‐incidence wide‐angle X‐ray scattering (GIWAXS) measurements were conducted at a SAXS system (SAXSSpoint 5.0) with a Pilatus3R 300K detector. Energy‐dispersive spectroscopy (EDS) images were obtained using a HITACHI SU8600 scanning electron microscope. Steady‐state photoluminescence (PL) and time‐resolved photoluminescence (TRPL) were measured at 804 nm using an excitation wavelength of 450 nm by a Spectrofluorometer FLS980 (Edinburgh Instruments Ltd.).

The transient photovoltage (TPV) and transient photocurrent (TPC) were recorded on a digital oscilloscope (Tektronix MDO3032). Green light pulse diodes (510 nm, 0.05 s square pulse width, 100 ns rise and fall time) controlled by a fast solid‐state switch were used as the perturbation source. The internal frequency of the oscilloscope was set to 20 kHz for TPV, and the internal impedance of the oscilloscope was set to 50 Ω for TPC measurements. Current density‐voltage (*J‐V*) characteristics of PSCs were measured by an Enlitech Solar Simulator SS‐X100A, which offered simulated AM 1.5G illumination (100 mW cm^−2^), and a Keysight B2901BL source meter. The intensity of the solar simulator was calibrated using a standard silicon cell before testing. The active areas of the devices are 0.049 cm^2^, which are defined by metal masks. The *J‐V* curves were measured using a reverse scan (from a forward bias (1.2 V) to short circuit (0 V)) and a forward scan (from a short circuit (1.2 V) to forward bias (0 V)). The step voltage was fixed at 100 mV. Stabilized power output measurements of the devices were obtained by maintaining a constant voltage near the maximum power point and recording the corresponding current output under illumination. External Quantum Efficiency (EQE) spectra were measured using the Enlitech QE‐R measurement system. The system was calibrated using a silicon reference cell with a known spectral response before measurement.

The operational stability of unencapsulated devices was tested using a multi‐channel solar cell stability test system (LDT‐3030, Jiangsu Datamaker Technology Co. Ltd). The operational aging test was performed under MPP tracking under continuous white LED array illumination, and the light intensity was calibrated to achieve the same current density as that of the standard silicon wafer solar cell measured under a standard solar simulator (AM1.5G sunlight, 100 mW cm^−2^). The tests were carried out in a nitrogen atmosphere, and the operating temperature was around 40°C.

[CCDC 2554494 contains the supplementary crystallographic data for this paper. These data can be obtained free of charge from The Cambridge Crystallographic Data Centre via www.ccdc.cam.ac.uk/data_request/cif.]

## Author Contributions

**Xinbao Lin**: investigation, data curation, methodology, Writing – original draft, formal analysis. **Xiangyang Zhang**: methodology, investigation, data curation, writing – original draft. **Guanghao Meng**: methodology, visualization, software. **Xiaozheng Zhao**: methodology. **Yanan Liu**: investigation. **Tianci An**: investigation. **Min Li**: validation. **Wenya Zhao**: investigation. **Miaoran Zhang**: investigation. **Linfeng Cui**: investigation. **Taoyu Weng**: writing – review and editing, investigation, validation, formal analysis, software. **Yantao Shi**: supervision, writing – review and editing, investigation, data curation, validation. **Bo Liu**: supervision, funding acquisition, writing – review and editing, conceptualization, investigation, visualization, project administration, resources.

## Funding

This work was supported by the International Science & Technology Cooperation Program of Hebei Province (25294402D), the Central Guiding Fund for Local Science and Technology Development of Hebei Province (264Z4301G), the Foreign Expert Category of the Top – notch Talent Project under the Yanzhao Golden Platform Talent – gathering Program in Hebei Province (Overseas Platform), the Hebei Natural Science Foundation (B2026205002), the Science Research Project of Hebei Education Department (QN2026476), the Science Foundation of Hebei Normal University (L2025B56), Spring City Plan: the High‐level Talent Promotion and Training Project of Kunming (2022SCP005), and the Yunnan Provincial Graduate Supervisor Team Construction Project (2024).

## Conflicts of Interest

The authors declare no conflicts of interest.

## Supporting information




**Supporting File 1**: advs78721‐sup‐0001‐SuppMat.docx.

## Data Availability

The data that support the findings of this study are available from the corresponding author upon reasonable request.
