# Supplementary material for: Molecular Engineering of Multi‐Site Small‐Molecule Chelation for Highly Efficient and Stable n‐i‐p Perovskite Solar Cells
Source: Adv Sci (Weinh). 2026 Nov 9:e78721. Online ahead of print. doi: 10.1002/advs.78721 (PMC13748895; doi:10.1002/advs.78721)
Supplement: Supplementary file 1 [file ADVS-9999-e78721-s001.docx]

Supporting Information

**Molecular Engineering of Multi-Site Small-Molecule Chelation for Highly Efficient and Stable n-i-p Perovskite Solar Cells**

Xinbao Lin, Xiangyang Zhang, Guanghao Meng, Xiaozheng Zhao, Yanan Liu, Tianci An, Min Li, Wenya Zhao, Miaoran Zhang, Linfeng Cui, Taoyu Weng,* Yantao Shi,* and Bo Liu*

X. Lin, X. Zhao, Y. Liu, T. An, M. Li, W. Zhao, M. Zhang, L. Cui, T. Weng, B. Liu

Hebei Technology Innovation Center for Energy Conversion Materials and Devices, Hebei Engineering Research Center of Thin Film Solar Cell Materials and Devices, National Demonstration Center for Experimental Chemistry Education, College of Chemistry and Material Science, Hebei Normal University, Shijiazhuang 050024, China
E-mail: taoyuweng@hebtu.edu.cn, [liubo@hebtu.edu.cn](mailto:liubo@hebtu.edu.cn)

X. Zhang, Y. Shi

State Key Laboratory of Fine Chemicals, School of Chemistry, Frontier Science Center for Smart Materials, Dalian University of Technology, Dalian 116024, China
E-mail: [shiyantao@dlut.edu.cn](mailto:shiyantao@dlut.edu.cn)

G. Meng

Yunnan Key Laboratory of Modern Separation Analysis and Substance Transformation, College of Chemistry and Chemical Engineering, Key Laboratory of Education Ministry for Advance Technique and Preparation of Renewable Energy Materials, Yunnan Normal University, Kunming 650500, China

Synthetic details

**Figure S1.** Synthesis routes of the three molecules.

Synthesis of **compound 1**：To a 100 mL round-bottomed flask, 4,4′-dimethoxydiphenylamine (288 mg, 1.26 mmol), 2-bromo-5-iodoanisole (492 mg, 1.57 mmol) and sodium tert-butoxide (189 mg, 1.89 mmol) were dissolved in anhydrous toluene (50 mL) and the mixture was deoxygenated with argon for 20 min. Then Pd(dba)_3_ (65 mg, 0.1 mmol) and Pd(dppf)Cl_2_ (56 mg, 0.1 mmol) were added to the reaction mixture, then purged again with argon for 20 min. The mixture was stirred at 120 ℃ for 12 h, and then cooled to room temperature. The mixture was washed with water and extracted with DCM. The combined organic layer was dried with anhydrous sodium sulfate, and the solvent was removed under reduced pressure. The residue was purified by column chromatography using hexane/ethyl acetate (200: 1) to give a white solid (473 mg, 72.6 % yield). ^1^H NMR (400 MHz, DMSO-*d*_6_, δ): 7.30 (d, *J* = 8.7 Hz, 1H), 7.04 (d, *J* = 8.9 Hz, 4H), 6.91 (d, *J* = 8.9 Hz, 4H), 6.43 (d, *J* = 2.6 Hz, 1H), 6.21 (dd, *J* = 8.7, 2.5 Hz, 1H), 3.73 (s, 6H), 3.59 (s, 3H).

Synthesis of **compound 2**：The compound 1 (500 mg, 1.20 mmol), bis(pinacolato)diboron (764 mg, 3.00 mmol), potassium acetate (1970 mg, 6.28 mmol), sodium tert-butoxide (756 mg, 7.56 mmol) and anhydrous toluene (50 mL) were added to a 100 mL round-bottomed flask. The mixture was deoxygenated with argon for 20 min. Then Pd(dppf)Cl_2_ (224 mg, 0.40 mmol) was added to the reaction mixture, then purged again with argon for 20 min. The mixture was stirred at 120 ℃ for 16 h, and then cooled to room temperature. The mixture was washed with water and extracted with DCM. The combined organic layer was dried with anhydrous sodium sulfate, and the solvent was removed under reduced pressure. The residue was purified by column chromatography using hexane/ethyl acetate (200: 1) to give compound 2 as a yellow oil (452 mg, 81.2 % yield). ^1^H NMR (400 MHz, DMSO-*d*_6_, δ): 7.34 (d, *J* = 8.1 Hz, 1H), 7.06 (d, *J* = 8.6 Hz, 4H), 6.92 (d, *J* = 8.7 Hz, 4H), 6.22 (d, *J* = 9.6 Hz, 1H), 3.74 (s, 6H), 3.47 (s, 3H), 1.22 (s, 12H).

Synthesis of **TPABT1**：In a round-bottomed flask, 4,7-dibromo-2,1,3-benzothiadiazole (400 mg, 1.36 mmol), 4-methoxy-N-(4-methoxyphenyl)-N-(4-(4,4,5,5-tetramethyl-1,3,2-dioxaborolan-2-yl)phenyl)aniline (1300 mg, 3.00 mmol) and cesium carbonate (260 mg, 0.80 mmol) were dissolved in toluene/H_2_O (50/5.00 mL) and the mixture was deoxygenated with argon for 20 min. Then Pd(dppf)Cl_2_ (120 mg, 0.14 mmol) was added to the reaction mixture, then purged again with argon for 20 min. The mixture was stirred at 120 ℃ for 14 h, and then cooled to room temperature. The mixture was washed with water and extracted with DCM. The combined organic layer was dried with anhydrous sodium sulfate, and the solvent was removed under reduced pressure. The residue was purified by column chromatography using DCM/hexane (2: 1) to give a dark red solid (1080 mg, 69.5 % yield). ^1^H NMR (400 MHz, Chloroform-*d*, δ): 7.80 (d, *J* = 8.7 Hz, 4H), 7.68 (s, 2H), 7.13 (d, *J* = 8.4 Hz, 8H), 7.04 (d, *J* = 8.3 Hz, 4H), 6.85 (d, *J* = 8.9 Hz, 8H), 3.81 (s, 12H). ^13^C NMR (100 MHz, Chloroform-*d*, δ): 156.17, 148.85, 140.66, 132.09, 129.77, 127.20, 127.09, 119.91, 114.82, 77.16, 55.61. HRMS-ESI (*m/z*): [M]^+^ Calcd. For (C_46_H_38_N_4_O_4_S): 742.2608, found: 742.2607

Synthesis of **TPABT2**: The synthesis process of TPABT2 is like TPABT1. The only difference is that 4,7-dibromo-2,1,3-benzothiadiazole changed to 4, 7-dibromo-5, 6-dimethoxybenzo[c][1, 2, 5]thiadiazole. The residue was purified by column chromatography using DCM/hexane (2: 1) to give a red solid (350 mg, 69.7 % yield). ^1^H NMR (400 MHz, Chloroform-*d*, δ): 7.53 (d, *J* = 8.3 Hz, 4H), 7.15 (d, *J* = 8.4 Hz, 8H), 7.02 (d, *J* = 8.3 Hz, 4H), 6.86 (d, *J* = 8.9 Hz, 8H), 3.80 (s, 18H). ^13^C NMR (100 MHz, Chloroform-*d*, δ): 156.19, 153.36, 148.49, 140.66, 131.15, 127.31, 124.81, 123.69, 118.98, 114.81, 61.47, 55.60. HRMS-ESI (*m/z*): [M]^+^ Calcd. For (C_48_H_42_N_4_O_6_S), 803.2820, found: 802.2818.

Synthesis of **TPABT3**: The synthesis process of TPABT3 is like TPABT2. The difference is that 4,7-dibromo-2,1,3-benzothiadiazole changed to compound 2 and the solvent changed to xylene. The residue was purified by column chromatography using DCM/hexane (1: 1) to give a yellow solid (330 mg, 45.5 % yield). ^1^H NMR (400 MHz, Acetone-*d*_6_, δ): 7.17 (t, *J* = 7.0 Hz, 10H), 6.96 (d, *J* = 8.9 Hz, 8H), 6.65 (s, 2H), 6.50 (d, *J* = 8.3 Hz, 2H), 3.81 (s, 12H), 3.77 (d, *J* = 2.7 Hz, 6H), 3.53 (d, *J* = 5.4 Hz, 6H). ^13^C NMR (100 MHz, Acetone-*d*_6_, δ): 157.72, 156.19, 154.08, 152.64, 150.08, 140.58, 131.84, 127.43, 120.72, 114.65, 111.79, 102.94, 61.07, 55.49. HRMS-ESI (*m/z*): [M]^+^ Calcd. For (C_50_H_46_N_4_O_8_S), 862.3031, found: 862.3028.

**DFT calculations**

The geometrical and electronic properties of TPABT and Li^+^ were conducted on the Gaussian 09 program using B31LYP as the exchange-correlation function, and 6-311G* basis sets with default spin mode. All structure geometries were fully optimized. After geometry optimization, a frequency calculation was performed to ensure the stability of the optimized structure. The binding energies (*E*_b_) of Li^+^ with TPABT were calculated by the following equation.

$$\text{E}\text{b}\text{ }\text{=}{\text{ }\text{E}}_{\text{Li}^{\text{+}}-\text{TPABT}}-\text{(}\text{E}_{\text{Li}^{\text{+}}}\text{ }\text{+}{\text{ }\text{E}}_{\text{TPABT}}\text{)}$$

Where $\text{E}_{\text{Li}^{\text{+}}-\text{TPABT}}$ is the total energy of Li^+^ in complex with TPABT, $\text{E}_{\text{Li}^{\text{+}}}$ means the energy of Li^+^ and the $\text{E}_{\text{TPABT}}$ stands for the energy of TPABT.

**Equations**

**Space-charge-limited-current (SCLC) analysis**

SCLC analysis was carried out on a hole-only device. Parameters of dark *J-V* curves of perovskite films (ITO/PTAA/perovskite/HTL/Ag). The defect densities (*N*_t_) are determined according to the equation:

*N*_t_ = 2*V*_TFL_*ε*_r_*ε*_0_/ (*ed*^2^)

The charge carrier mobility (*μ*_h_) in device films can be estimated based on the measured *J-V* curves following Mott-Gurneyʼs square law:

*J*_D_ = 9*με*_r_*ε*_0_*V*_b_^2^/8*d*^2^

where *ε*_0_ is the vacuum permittivity, *ε*_r_ is the relative dielectric constant, V_TFL_ represents the trap-filled limit voltage, *e* is the elementary charge, *J*_D_ denotes the dark current density, *V*_b_ is the applied bias voltage, and *d* is the film thickness.

**Urbach energy** **analysis**

In accordance with the Urbach rule, the correlation between the absorption coefficient and the photon energy in the localised state can be expressed by an exponential function:

$$\alpha=\frac{\alpha_{\text{0}}exp(hv-\text{E}_{\text{0}}\text{)}}{\text{E}_{\text{u}}}$$

where *α_0_* is a constant, *E_0_* is the energy threshold associated with the material properties, *E_u_* is the Urbach energy, *α* is the material absorption coefficient, and *hv* is the relationship between photon energies. Urbach energy is obtained by linear fitting of ln(*α*) vs photon energy relationship.

**The hysteresis index (*H*-index)**

*H*-index = (PCE_reverse_ − PCE_forward_)/PCE_reverse_

***V*_OC_ -light intensity analysis**

A linear relationship between *Voc* and the logarithm of light intensity was found for all samples, evidenced by the straight fitting lines. According to the equation:

$$Voc=\frac{\text{nT}\text{k}_{\text{B}}}{\text{q}}\text{ ln (}\frac{\text{I}\text{+}\text{I}_{\text{0}}}{\text{I}_{\text{0}}}\text{)}$$

where $\text{k}_{\text{B}}$ denotes the Boltzmann constant, $\text{T}$ represents the absolute temperature, and $\text{q}$ is the electric charge.

**Fill factor (*FF*) loss analysis**

According to the S-Q limit theory, non-radiative recombination losses and charge transport losses cause the measured *FF* to be lower than the theoretical value. Generally, the lower the non-radiative recombination loss of the device, the higher its maximum *FF* (*FF*_max_) value. The *FF*_max_ can be calculated using the following equation:

$$FFmax=\frac{\mathcal{V}_{\text{OC}}\text{-ln(}\mathcal{V}_{\text{OC}}+0.72)}{\mathcal{V}_{\text{OC}}\text{+1}}$$

The $\mathcal{V}_{\text{OC}}$ can be obtained by the following equation:

$$\mathcal{V}_{\text{OC}}=\frac{V_{\mathrm{OC}}}{\text{nT}\text{k}_{\text{B}}/q}$$

where n is the ideality factor extracted from the slope of the *V*_OC_-light dependence curve.

**Figures**

Figure S2. ESP distribution maps of TPABT1-3.

**Figure S3.** Solid-state ^7^Li and ^1^H NMR of Li-TFSI and TPABT3+Li-TFSI mixture.

Figure S4. Mulliken charge distribution of atoms in TPABT3 without and with binding Li^+^ ions.

**Figure S5.** ^7^Li and ^1^H NMR of the Li-TFSI solutions without and with TPABT1-2. The solvents used for TPABT1 were deuterated acetone, while deuterated DMSO was used for TPABT2.

Figure S6. XPS spectra (Li 1s) of control and doped Spiro-OMeTAD films.

Figure S7. In situ UV-vis absorption spectra of control and Spiro+TPABT3.

Figure S8. Binding energy of Spiro-OMeTAD‒Spiro-OMeTAD and Spiro-OMeTAD‒TPABT3 calculated using density functional theory.

Figure S9. EDS images of perovskite/doped Spiro-OMeTAD films.

Figure S10. The Urbach energy (*E*_u_) of control and doped HTL/PVK samples.

Figure S11. The crystal data of TPABT3.

**Figure S12.** EPR results of (a) Spiro-OMeTAD+TPABT molecules (without Li-TFSI) and (b) TPABT+Li-TFSI (without Spiro-OMeTAD).

**Figure S13**. UV-vis absorption spectra of the individual TPABT1-3 solutions.

F**igure S14**. UV-vis absorption spectra of the TPABT+Li-TFSI solutions.

**Figure S15.** EPR results of Spiro+TPABT3 solution (without O_2_).

**Figure S16.** UV-vis absorption spectra of Spiro+TPABT3 solution (without O_2_).

**Figure S17.** Comparison of the color changes of control and doped Spiro-OMeTAD solutions before and after aging for 5 hours (without O_2_).

Figure S18. The carrier concentration of control and doped Spiro-OMeTAD films from Hall measurement.

Figure S19. UPS spectra of perovskite and different doped Spiro-OMeTAD films.

Figure S20. UV-vis absorption spectra of perovskite (a) and HTL films(b). Tauc plots of perovskite (c) and HTL films (d).

Figure S21. *J-V* characteristic curves of PSCs with different concentrations of TPABT molecules.

Figure S22. *J-V* curves of PSCs measured in forward scanning and reverse scanning.

Figure S23. The statistical data of (a) *V*oc, (b) *J*sc, (c) FF, and (d) PCE obtained from control and doped devices.

Figure S24. Stable power output of PSCs.

Figure S25. The light intensity-dependent *V*oc curves of control and doped devices.

Figure S26. TPV decay curves of control and doped devices.

Figure S27. The light intensity-dependent *J*sc curves of control and doped devices.

Figure S28. Space-charge-limited current characteristics of the devices with an architecture of ITO/PTAA/perovskite/control or doped HTLs/Au.

Figure S29. Nyquist plots of control and doped devices.

Figure S30. The moisture stability of devices. (85% RH).

Figure S31. The water contact angle (WCA) of the control and doped Spiro-OMeTAD films.

Figure S32. The thermal stability of devices at 85℃.

Figure S33. ^1^H NMR spectra of compound 1 (DMSO-*d_6_*).

Figure S34. ^1^H NMR spectra of compound 2 (DMSO-*d_6_*).

Figure S35. ^1^H NMR spectra of TPABT1 (Chloroform-*d*).

Figure S36. ^13^C NMR spectra of TPABT1 (Chloroform-*d*).

Figure S37. The high-resolution mass spectra of TPABT1.

Figure S38. ^1^H NMR spectra of TPABT2 (Chloroform-*d*).

Figure S39. ^13^C NMR spectra of TPABT2 (Chloroform-*d*).

Figure S40. The high-resolution mass spectra of TPABT2.

Figure S41. ^1^H NMR spectra of TPABT3 (*d*-Acetone).

Figure S42. ^13^C NMR spectra of TPABT3 (*d*-Acetone).

Figure S43. The high-resolution mass spectra of TPABT3.

**Tables**

**Table S1.** Single-crystal data of TPABT3.

| Empirical formula | C_50_H_46_N_4_O_8_S |
| --- | --- |
| Formula weight | 862.97 |
| Temperature/K | 173.00 |
| Crystal system | triclinic |
| Space group | P1 |
| a/Å | 9.1372(9) |
| b/Å | 10.6459(8) |
| c/Å | 15.0627(15) |
| α/° | 83.941(7) |
| β/° | 72.537(7) |
| γ/° | 69.555(7) |
| Volume/Å^3^ | 1309.6(2) |
| Z | 1 |
| ρ_calc_g/cm^3^ | 1.094 |
| μ/mm^‑1^ | 0.964 |
| F (000) | 454.0 |
| Crystal size/mm^3^ | 0.3 × 0.1 × 0.1 |
| Radiation | CuKα (λ = 1.54178) |
| 2Θ range for data collection/° | 6.15 to 137.642 |
| Index ranges | -9 ≤ h ≤ 11, -12 ≤ k ≤ 12, -16 ≤ l ≤ 17 |
| Reflections collected | 18603 |
| Independent reflections | 5770 [Rint = 0.0376, Rsigma = 0.0462] |
| Data/restraints/parameters | 5770/816/723 |
| Goodness-of-fit on F^2^ | 1.425 |
| Final R indexes [I>=2σ (I)] | R_1_ = 0.1038, wR_2_ = 0.3072 |
| Final R indexes [all data] | R_1_ = 0.1323, wR_2_ = 0.3418 |
| Largest diff. peak/hole / e Å^-3^ | 0.34/-0.30 |
| Flack parameter | 0.47(3) |
| *CCDC* number | 2554494 |

**Table S2.** Energy level parameters of PVK, Spiro-OMeTAD, and doped Spiro-OMeTAD films.

| **Sample** | **Onset [eV]** | **Cutoff [eV]** | ***E*_f_ [eV]** | **VBM [eV]** | **CBM [eV]** |
| --- | --- | --- | --- | --- | --- |
| PVK | 0.84 | 16.68 | -4.54 | -5.38 | -3.82 |
| Control | 1.02 | 17.07 | -4.15 | -5.17 | -2.19 |
| Spiro+TPABT1 | 0.89 | 16.92 | -4.30 | -5.19 | -2.21 |
| Spiro+TPABT2 | 0.90 | 16.89 | -4.33 | -5.23 | -2.25 |
| Spiro+TPABT3 | 0.91 | 16.86 | -4.36 | -5.27 | -2.30 |

**Table S3.** Parameters of TRPL spectra fitted by a two-phase exponential decay function f(t) = A_1_exp(−t/*τ*_1_) + A_2_exp(−t/*τ*_2_).

| **Sample** | **A_1_ [%]** | ***τ*_1_ [ns]** | **A_2_ [%]** | ***τ*_2_ [ns]** | **τ_ave_ [ns]** |
| --- | --- | --- | --- | --- | --- |
| PVK | 67.16 | 368.85 | 32.84 | 1676.83 | 1271.23 |
| PVK/Control | 52.59 | 55.32 | 47.41 | 1002.24 | 947.61 |
| PVK/Spiro+TPABT1 | 51.76 | 50.95 | 48.24 | 778.17 | 730.44 |
| PVK/Spiro+TPABT2 | 66.23 | 60.01 | 33.77 | 518.79 | 433.96 |
| PVK/Spiro+TPABT3 | 59.78 | 65.55 | 40.32 | 397.72 | 332.49 |

**Table S4.** The photovoltaic parameters of PSCs are based on different doped HTL with 2, 5, and 10 % mol concentration.

| **Devices** | ***V*oc [V]** | ***J*sc [mA/cm^−2^]** | **FF [%]** | **PCE [%]** |
| --- | --- | --- | --- | --- |
| Spiro+TPABT1 (2% mol) | 1.16 | 25.42 | 78.56 | 23.16 |
| Spiro+TPABT1 (5% mol) | 1.16 | 26.13 | 81.64 | 24.79 |
| Spiro+TPABT1 (10% mol) | 1.14 | 25.48 | 71.37 | 20.73 |
| Spiro+TPABT2 (2% mol) | 1.16 | 25.57 | 79.78 | 23.56 |
| Spiro+TPABT2 (5% mol) | 1.16 | 26.24 | 83.35 | 25.42 |
| Spiro+TPABT2 (10% mol) | 1.15 | 25.13 | 76.84 | 21.95 |
| Spiro+TPABT3 (2% mol) | 1.17 | 25.91 | 81.51 | 24.62 |
| Spiro+TPABT3 (5% mol) | 1.17 | 26.39 | 84.08 | 26.03 |
| Spiro+TPABT3 (10% mol) | 1.16 | 25.44 | 78.35 | 23.17 |

**Table S5.** The photovoltaic parameters of champion devices based on control and doped Spiro-OMeTAD under reverse scan and forward scan.

| **Devices** | ***V*oc [V]** | ***J*sc [mA/cm^−2^]** | **FF [%]** | **PCE [%]** | **HI [%]** |
| --- | --- | --- | --- | --- | --- |
| Spiro+TPABT3 Reverse | 1.17 | 26.39 | 84.08 | 26.03 | 0.4% |
| Spiro+TPABT3 Forward | 1.17 | 26.51 | 83.51 | 25.92 |  |
| Spiro+TPABT2 Reverse | 1.16 | 26.24 | 83.35 | 25.42 | 1.2% |
| Spiro+TPABT2 Forward | 1.16 | 26.19 | 82.47 | 25.12 |  |
| Spiro+TPABT1 Reverse | 1.16 | 26.13 | 81.64 | 24.79 | 1.8% |
| Spiro+TPABT1 Forward | 1.16 | 26.03 | 80.52 | 24.33 |  |
| Spiro-OMeTAD Reverse | 1.16 | 25.85 | 79.45 | 23.85 | 2.3% |
| Spiro-OMeTAD Forward | 1.15 | 25.78 | 78.27 | 23.29 |  |

**Table S6.** The photovoltaic parameters of champion two-step formamidinium-based n-i-p devices in this work and recent literature of HTL engineering.

| **Devices** | ***V*oc [V]** | ***J*sc [mA/cm^−2^]** | **FF [%]** | **PCE [%]** | **Ref.** |
| --- | --- | --- | --- | --- | --- |
| DDT | 1.198 | 25.90 | 79.20 | 24.60 | [1] |
| BDT-C8-3O | 1.163 | 25.51 | 81.31 | 24.11 | [2] |
| IP-TFSI | 1.190 | 25.07 | 84.35 | 25.16 | [3] |
| Spiro-BD-T | 1.170 | 24.90 | 82.90 | 24.20 | [4] |
| PC | 1.181 | 25.52 | 82.26 | 24.80 | [5] |
| MSBH | 1.190 | 26.31 | 80.92 | 25.30 | [6] |
| PBTTT-C_14_ | 1.180 | 25.49 | 84.35 | 25.37 | [7] |
| 5D-Spiro-OMeTAD | 1.187 | 26.65 | 81.08 | 25.65 | [8] |
| TCPO | 1.187 | 25.91 | 83.50 | 25.68 | [9] |
| oil-NiOx NPs | 1.183 | 25.34 | 81.00 | 24.20 | [10] |
| E-Spiro-OMeTAD | 1.187 | 26.18 | 84.20 | 26.16 | [11] |
| Mg^2+^/Ca^2+^ | 1.146 | 25.30 | 82.78 | 24.20 | [12] |
| TFMP | 1.204 | 25.56 | 84.20 | 25.92 | [13] |
| TPABT3 | 1.173 | 26.39 | 84.08 | 26.03 | This work |

**References**

1.	X. Liu, B. Zheng, L. Shi, et al., “Perovskite solar cells based on spiro-OMeTAD stabilized with an alkylthiol additive,” *Nature Photonics* 17 (2022): 96-105.

<https://doi.org/10.1038/s41566-022-01111-x>

2.	Q. Cheng, H. Chen, W. Chen, et al., “Green solvent processable, asymmetric dopant‐free hole transport layer material for efficient and stable n‐i‐p perovskite solar cells and modules,” *Angewandte Chemie International Edition* 62 (2023): e202312231.

<https://doi.org/10.1002/anie.202312231>

3	H. Yang, T. Xu, W. Chen, et al., “Iodonium initiators: paving the air‐free oxidation of spiro‐OMeTAD for efficient and stable perovskite solar cells,” *Angewandte Chemie International Edition* 63 (2023): e202316183.

<https://doi.org/10.1002/anie.202316183>

4	H. Yang, Y. Shen, G. Xu, et al., “Functional spiro-OMeTAD-like dopant for Li-ion-free hole transport layer to develop stable and efficient n-i-p perovskite solar cells,” *Nano Energy* 119 (2024): 109033.

<https://doi.org/10.1016/j.nanoen.2023.109033>

5.	R. Zhao, T. Wu, R. Huang, Y. Wang, and Y. Hua, “A universal and versatile hole transport layer “activator” enables stable perovskite solar cells with efficiency near 25%,” *Advanced Functional Materials* 34 (2024): 2314758.

<https://doi.org/10.1002/adfm.202314758>

6.	J. Xu, P. Shi, K. Zhao, et al., “Ion-migration inhibitor for spiro-OMeTAD/perovskite contact toward stable perovskite solar cells,” *ACS Energy Letters* 9 (2024): 1073-1081.

<https://doi.org/10.1021/acsenergylett.4c00049>

7.	T. Zhao, X. Jin, M.-H. Li, et al., “π-Conjugation-induced in situ nanoscale ordering of spiro-OMeTAD boosts the efficiency and stability of perovskite solar cells,” *Journal of the American Chemical Society* 146 (2024): 30893-30900.

<https://doi.org/10.1021/jacs.4c09094>

8.	S. Wang, S. Zhang, X. Shi, et al., “Self-cleaning spiro-OMeTAD via multimetal doping for perovskite photovoltaics,” *Nature Communications* 16 (2025): 4167.

<https://doi.org/10.1038/s41467-025-59350-9>

9.	Q. Zhou, Y. Yang, D. He, et al., “Simultaneous suppression of multilayer ion migration via molecular complexation strategy toward high‐performance pegular perovskite solar cells,” *Angewandte Chemie International Edition* 64 (2024): e202416605.

<https://doi.org/10.1002/anie.202416605>

10.	W. Zhang, L. Cojocaru, H. Tamegai, et al., “NiOx nanoparticles as p-dopants for instant oxidation of spiro-OMeTAD enabling high-performance and stable perovskite solar cells,” *Nano Energy* 146 (2025): 111499.

<https://doi.org/10.1016/j.nanoen.2025.111499>

11.	H. Huang, Z. Lan, Y. Yang, et al., “Controllable electrolysis doping of organic semiconductors for stable perovskite solar cells,” *Joule* 9 (2025): 102106.

<https://doi.org/10.1016/j.joule.2025.102106>

12.	L. Hu, X. Qi, R. Zhao, et al., “Modulating janus effect of cations to control electronic symmetry and hole mobility in spiro‐OMeTAD for perovskite solar cells,” *Angewandte Chemie International Edition* 65 (2025): e21967.

<https://doi.org/10.1002/anie.202521967>

13.	Y. Kim, K. M. Yeom, H. Kim, et al., “Controlling doping preference of spiro‐OMeTAD for efficient and stable perovskite solar cells,” *Advanced Energy Materials* 16 (2026): e06760.

<https://doi.org/10.1002/aenm.202506760>
